# Correction: Investigation of plasma metabolomics and neurotransmitter dysfunction in the process of Alzheimer’s disease rat induced by amyloid beta 25-35

**DOI:** 10.1039/d1ra90081a

**Published:** 2021-02-15

**Authors:** Mengying Wei, Yuanyuan Liu, Zifeng Pi, Kexin Yue, Shizhe Li, Mingxin Hu, Zhiqiang Liu, Fengrui Song, Zhongying Liu

**Affiliations:** School of Pharmaceutical Sciences, Jilin University 1266 Fujin Road Changchun 130021 China liuzy@jlu.edu.cn +86 431 85619704; National Center for Mass Spectrometry in Changchun, Jilin Province Key Laboratory of Chinese Medicine Chemistry and Mass Spectrometry, Changchun Institute of Applied Chemistry, Chinese Academy of Sciences Changchun 130022 China; Guangdong Univ Technol, Inst Biomed & Pharmaceut Sci Guangzhou 510006 Guangdong People’s Republic of China

## Abstract

Correction for ‘Investigation of plasma metabolomics and neurotransmitter dysfunction in the process of Alzheimer’s disease rat induced by amyloid beta 25-35’ by Mengying Wei *et al.*, *RSC Adv.*, 2019, **9**, 18308–18319. DOI: 10.1039/C9RA00302A.

The authors regret that, due to personal negligence, Fig. 1d-NG-4 in this article was repeatedly uploaded as Fig. 1d-NG-2. The authors apologize to readers for this inaccuracy and the corrected figure is shown below. This correction does not affect the results, discussion or conclusion of the article.

**Fig. 1:**
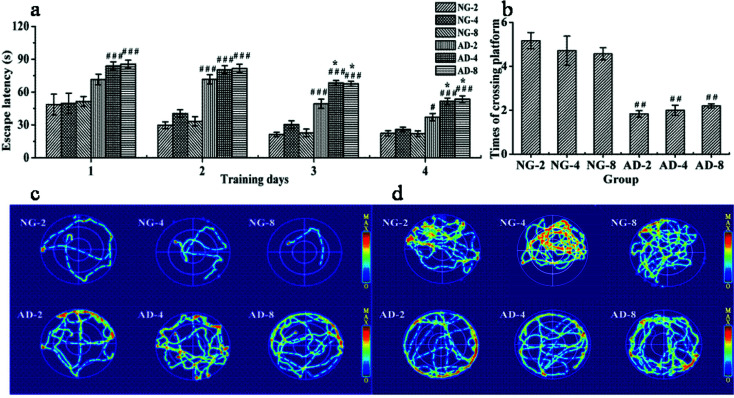
The performance of spatial learning and memory in rats after 2, 4 and 8 weeks of modelling in the MWM test: (a) the escape latency during the 4 day training period, (b) times of crossing the original platform in the 120 s probe test, (c) trajectories of the last trial, (d) trajectories of rats from each group in (b). Notes: *n* = 10, per group; data are expressed as mean ± SEM, compared to NG by a *t*-test after the same week, ^###^*P* < 0.001, ^##^*P* < 0.01, ^#^*P* < 0.05, compared to AD-2, ***P < 0.001, **P < 0.01, *P < 0.05, compared to AD-4, ^&&&^*P* < 0.001, ^&&^*P* < 0.01, ^&^*P* < 0.05.

The Royal Society of Chemistry apologises for these errors and any consequent inconvenience to authors and readers.

## Supplementary Material

